# MGtree: A Fast and Flexible Alignment-Based Metagenomics Pipeline

**DOI:** 10.3390/v18060643

**Published:** 2026-06-03

**Authors:** Samantha L. Sholes, Scott Norton, Alfredo Gonzalez, John M. Gaspar

**Affiliations:** 1 Department of Data Science & Scientific Informatics, MRL—Information Technology, Merck & Co., Inc., West Point, PA 19486, USA; samantha.sholes@merck.com; 2 Department of Data Science & Scientific Informatics, MRL—Information Technology, Merck & Co., Inc., Cambridge, MA 02141, USA

**Keywords:** metagenomics, phylogenetics, short read alignment, norovirus, HPV

## Abstract

Metagenomics analysis is a critical tool in identifying and typing viral samples to aid surveillance, clinical, epidemiological, and other workflows. Despite advances in sequencing technology and analysis pipelines, there are still limitations that lead to reduced taxonomic resolution or false positives from highly recombinant or challenging samples. Here we describe MGtree, a novel metagenomics pipeline that utilizes a combination of full-length read alignments and phylogenetic analysis to classify samples of interest. We demonstrate that MGtree accurately genotypes viral samples from challenging norovirus and HPV datasets. MGtree outperforms the popular metagenomics programs Kraken2 and Centrifuge, and it succeeds with low-input samples where de novo assembly fails. MGtree’s correct assignments across highly mutant and coinfected samples highlights its ability to resolve viral genotypes and its potential to improve classification precision in complex samples.

## 1. Introduction

Metagenomics has vastly improved our understanding of complex microbial samples and ecosystem dynamics through the analysis of genetic material directly from the environment. With the high-throughput nature of next-generation sequencing (NGS), researchers can explore microbial communities without the need for culturing, enabling applications that range from assessing biodiversity in ecosystems to tracking infectious diseases [1,2]. In the latter case, the typing of viruses in biological samples is critical for identifying and monitoring viral prevalence, evolution, and fitness, and has significant implications for disease transmission, vaccine development, and treatment strategies [3].

Current metagenomics analysis tools allow researchers to identify and classify organisms across taxonomic levels. A popular program, Kraken2 [4], employs an exact k-mer matching approach to classify sequences against a reference database extremely rapidly, making it effective for dissecting complex microbial communities. However, the k-mer approach is inherently limited when one is seeking a more precise level of classification. When querying against reference sequences that are very similar, k-mers are more likely to be ambiguous; in samples that exhibit rapid evolution or contain novel genotypes, k-mers may not have exact matches. These limitations are exacerbated by the rapid growth in reference databases [5].

Another commonly used program, Centrifuge, uses a compressed FM-index to find near-exact matches to reference sequences rather than breaking reads into fixed k-mers [6]. This approach can account for shorter or partial matches, so it can be more tolerant of sequence divergence than a k-mer approach. However, there remains reduced sensitivity for reads that lack sufficiently long exact matches and for strains with high sequence identity across genomes, often leading to reduced classification resolution or false positives from conserved regions [7].

A third approach is to assemble the genome of the infectious agent de novo from the NGS reads by a program such as metaSPAdes [8] and then compare it to other known genomes. The assembly process is particularly computationally intensive and requires substantial sequence data [8,9]. An additional but critical step is to remove contaminating reads from other organisms, such as a human host, which can adversely affect the quality of the assembly [10]. Coinfections can also complicate the assembly process, as assemblies can be spread over multiple contigs and can be biased toward more abundant taxa. Once the assembly is completed, there are a number of convenient web-based tools for classification, such as Genome Detective [11] and more specialized databases [12,13], but using these tools can be problematic for those handling proprietary or clinical datasets.

Here we introduce MGtree, a fast, reliable, Nextflow-wrapped pipeline that utilizes a combination of full-length read alignments and phylogenetic analysis to classify samples of interest. By focusing on full-length alignments, the program ensures maximal use of the sequence information and assignment confidence even in samples with closely related sequences, coinfections, or other complex microbial interactions. MGtree allows users to provide custom reference sequences as well as taxonomic labels at which to report results. We benchmark MGtree on three previously published datasets and demonstrate that it matches results by orthogonal methods (PCR, Sanger sequencing). We also show that MGtree outperforms Kraken2, Centrifuge, and a de novo assembly-based approach.

## 2. Materials and Methods

### 2.1. MGtree Pipeline

**Figure 1:**
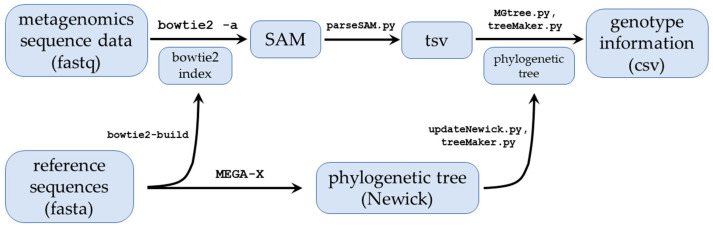
Schematic of the MGtree pipeline workflow from input fastq reads to reported genotype results. The python scripts of MGtree v1.0 are accompanied by two suggested programs, MEGA-X and bowtie2, for phylogenetic tree generation and short read alignment, respectively. The Nextflow wrapped branch of the pipeline includes batch processing of samples from fastq alignment with bowtie2 through the generation of genotype information.

The MGtree pipeline performs metagenomics classification of next-generation sequence reads against a set of reference sequences via a phylogenetic tree (Figure 1). The analysis in MGtree is alignment-based, and, to resolve ambiguities from closely related references, it works best when all valid alignments of a read/fragment are available to be analyzed, such as with bowtie2 [14] in -a mode. MGtree filters the alignments, keeping the primary ones, as well as secondary alignments that are equivalent (or within a user-specified threshold) based on alignment scores. The filtered alignments are then analyzed against a phylogenetic tree of the reference sequences. Counts are added to the tree at the lowest common ancestor (LCA) node of each set of alignments. The pipeline then interprets the LCA counts to produce an accounting of the constituents of the sample.

**Figure 2:**
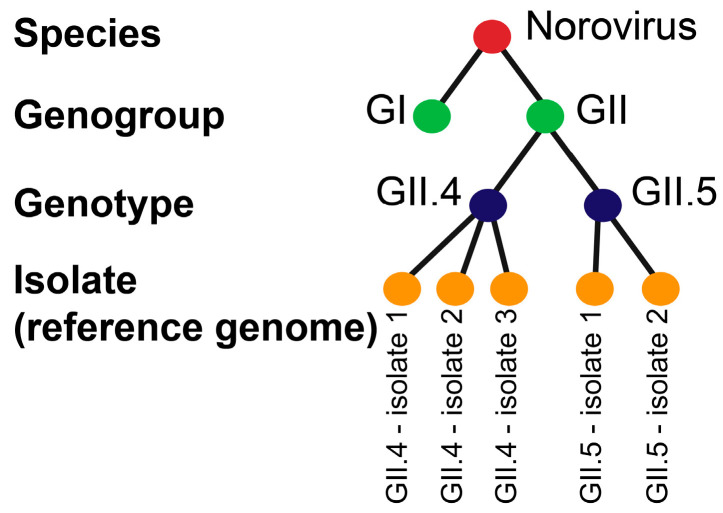
An example, minimal schematic of a norovirus phylogenetic tree. Reference genomes are shown as leaf nodes; internal nodes may be labeled, such as those with “Genotype” or “Genogroup” designations in this example. Reads are assigned to the lowest common ancestor node.

MGtree requires a phylogenetic tree of the reference sequences, created by a program such as MEGA-X [15]. One may wish to label internal nodes of the tree, such as those given “Genogroup” or “Genotype” designations in the toy example of a phylogenetic tree of norovirus sequences shown in Figure 2. The internal labels are used to guide the interpretation of the LCA counts; those below a labeled node are summed for that label, while those above a labeled node are reported as ambiguous among labeled descendants.

For example, a read aligning exclusively to “GII.4-isolate 1” (Figure 2) would be counted at that leaf node, and this would ultimately be reported as genotype GII.4 (if one were seeking output at the genotype level). A sample derived from a coinfection of GII.4 and GII.5 would consist of reads that may align to multiple references within those genotypes, but whose LCA nodes would be mostly at the genotype level. In this case, both genotypes would be reported in the output, along with their relative abundances. If there were any reads derived from a region of high homology between GII.4 and GII.5, those reads may align equally well to several reference sequences under both genotypes. For those reads, the LCA node would be the GII genogroup node, and the reads would be classified as ambiguous between the two genotypes.

The MGtree pipeline comes with a Nextflow (v24.04.2+) wrapper for the convenient and robust batch processing of a set of samples [16,17]. The Nextflow pipeline includes alignment with bowtie2, but note that the core scripts of MGtree can analyze any valid SAM file, including one produced via alignment of long reads. Additional details about MGtree can be found in Supplementary Note S1.

### 2.2. Benchmarking

MGtree v1.0 was benchmarked against Kraken2 v2.1.2, Centrifuge v1.0.4.2, and a de novo assembly approach (metaSPAdes v4.2.0 and virus-specific typing websites) on three previously published datasets. For all datasets, MGtree was run with default parameters. Kraken2 was run with a custom database and parameters for paired-end sequencing (--paired, k = 35, and ℓ = 31); we varied values of the --confidence and --minimum-hit-groups parameters, but the default values produced the best genotype-level results, which are reported here. Centrifuge was run with a custom database and --min-hitlen = 50; lower --min-hitlen values resulted in worse genotype results. Both Kraken2 and Centrifuge were also tested with pre-built indices (“Viral” for Kraken2 and “Refseq: bacteria, archaea, viral, human” for Centrifuge) but this resulted in fewer correctly reported genotypes. MetaSPAdes was run with the --meta or --metaviral modes to select for the most complete contigs after the removal of human reads using bowtie2 v2.3.5.1 and the hg38 reference genome [8,18]. Additional details and commands are provided in Supplementary Note S2.

#### 2.2.1. Norovirus

We analyzed thirty-four Illumina paired-end whole genome sequenced (WGS) norovirus samples taken from a study of fifteen outbreaks [19] (Supplementary Table S1). In that study, the genotypes of the viruses were identified by RT-qPCR and Sanger sequencing; in our benchmarking, we consider these genotypes to be ground truth.

**Figure 3:**
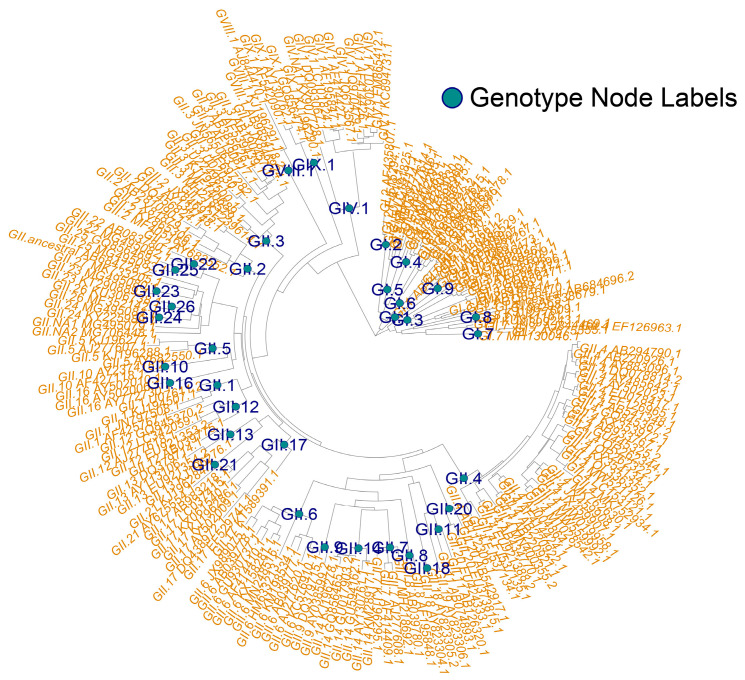
Norovirus phylogenetic tree with 204 isolate reference genomes. Internal nodes representing norovirus genotypes are labeled.

We generated a custom collection of 204 norovirus reference genomes with complete VP1 sequences (ORF2, genotyping region) from the CDC CaliciNet database [20] (Supplementary Table S2). We used MEGA-X v10.0.4 [15] to create a phylogenetic tree from these sequences, and then we ran the updateNewick.py script in MGtree to label the resulting phylogenetic tree at the genotype and genogroup levels (Figure 3). We also built a custom Kraken2 database and a Centrifuge database from the 204 norovirus genomes with kraken2-build and centrifuge-build, respectively, using the NCBI taxonomy library [4,6].

Assemblies generated by metaSPAdes were manually uploaded into the Calicivirus typing tool (accessed 12 May 2026) [12,13], which classifies based on the ORF2 sequence of the norovirus genome. We additionally uploaded the assemblies to Genome Detective v2.24.1 [11], but this produced the same results.

#### 2.2.2. HPV

We analyzed two datasets of HPV samples that had been genotyped by qPCR. The first contained seven Illumina paired-end WGS virally infected cancer cell lines (SiHa, HeLa, CaSki, C-33A, DoTc2, 2A3, and SCC154) [21]. The second consisted of twenty-nine Illumina paired-end WGS cervical cancer samples [22] (Supplementary Table S1).

We retrieved 224 HPV genomes from the PaVE database [23]. We generated a phylogenetic tree, labeling nodes at the HPV genotype level, and we created Kraken2 and Centrifuge databases as described above. Assemblies generated by metaSPAdes were manually uploaded into the PaVE BLAST typing tool (accessed 12 May 2026) [13].

## 3. Results

We used MGtree to analyze samples from three previously published datasets and evaluated its results against the samples’ genotypes that had been verified by orthogonal methods. We also compared MGtree’s results against those produced by the widely used metagenomics programs Kraken2 and Centrifuge, and a de novo assembly approach using the metaSPAdes assembler and viral-specific classification websites, on the same datasets.

The three published datasets were from one study of norovirus and two of HPV. Norovirus sequencing datasets are challenging to classify because the virus evolves rapidly and has a high recombination rate [24,25,26]. In addition, multiple reference sequences exist for each genotype (Figure 3). HPV frequently occurs as coinfections of multiple types, and there are extended regions of high sequence homology across multiple types’ genomes [27,28]. These complementary challenges presented by norovirus and HPV provide pertinent test cases of MGtree’s classification resolution.

### 3.1. Norovirus

**Figure 4:**
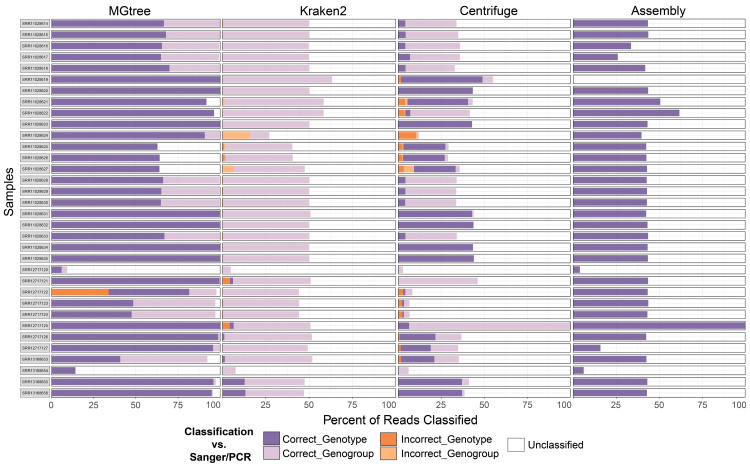
Norovirus typing by MGtree, Kraken2, Centrifuge, and de novo assembly with metaSPAdes and the Calicivirus typing tool. The percentage of norovirus WGS stool sample reads classified at the genotype and genogroup level by the four methods are colored by concordance with Sanger Sequencing and qPCR. Note that the assembly approach is classified only to the genotype level.

We evaluated MGtree on a WGS norovirus dataset derived from stool samples [19], containing a variety of norovirus genotypes (Supplementary Figure S1a). MGtree achieved an average correct genotype classification rate of 75.3% across samples, and it correctly classified more than 90% of the reads at the genotype level in nearly half of the samples (Figure 4). Some samples reached close to complete classification, such as SRR11028619 (99.9%) and SRR11028620 (99.8%). Most of the remaining reads were placed at the higher genogroup level correctly for each sample.

Only one sample, SRR127122, had more than 1% of reads reported as the incorrect genotype by MGtree. Although 47.8% of this sample’s reads were classified as the sample’s confirmed genotype, GII.3, 33.9% of them were called GII.4. This was unlikely due to a coinfection, since there was zero read coverage of GII.4 over most of the genome (Supplementary Figure S1b). Instead, in ORF1, there were spikes in read coverage in GII.4, many of which matched dips in coverage in GII.3, suggesting that this GII.3 norovirus sample had an ORF1 sequence that more closely matched that of GII.4 in certain locations. If we had limited our reference sequences to the canonical norovirus genotyping region (ORF2 only), MGtree would have classified the sample unambiguously as GII.3, but without the additional insight about the similarity of this sample’s ORF1 to GII.4.

In contrast to the classification rate of MGtree, Kraken2 classified an average of only 3.3% of the reads correctly at the genotype level (Figure 4), with most samples below 1%. Even at the higher genogroup level, its accuracy was only 44.4%. Kraken2 also exhibited a higher incidence of false positives, particularly at the genotype level, where fifteen samples had incorrect genotype assignments despite low percentages of classified reads. At the genogroup level, Kraken2 also showed some misclassifications, including as high as 15.8% incorrect assignments in sample SRR11028624. When adjusting the Kraken2 --confidence parameter, the rate of misclassification decreased, but with a loss of classified reads.

While Centrifuge outperformed Kraken2 at the genotype level, it still classified an average of only 17.2% of reads correctly. Centrifuge also exhibited a similar incidence of false positives to Kraken2; eleven samples had more than 1% of reads reported as incorrect genotypes, and two samples had more than 1% of reads reported as the incorrect genogroup (Figure 4). The rate of misclassification decreased with the adjustment of the --min-hitlen parameter, but this again caused a loss of classified reads.

The de novo assembly approach of metaSPAdes and the Calicivirus typing tool utilized an average of 40.2% of reads in correctly classified contigs, with no incorrect genotypes. Note that the read counts supporting each assembled contig were not reported directly, so we had to calculate these totals based on each contig’s coverage and size (see Supplementary Note S2 for details). For one sample, SRR11028619, assembly was unsuccessful, due to the limited number of norovirus reads (294 as reported). Assembly also required the most computational time and memory of the benchmarked pipelines, by far (Supplementary Table S3).

### 3.2. HPV

**Figure 5:**
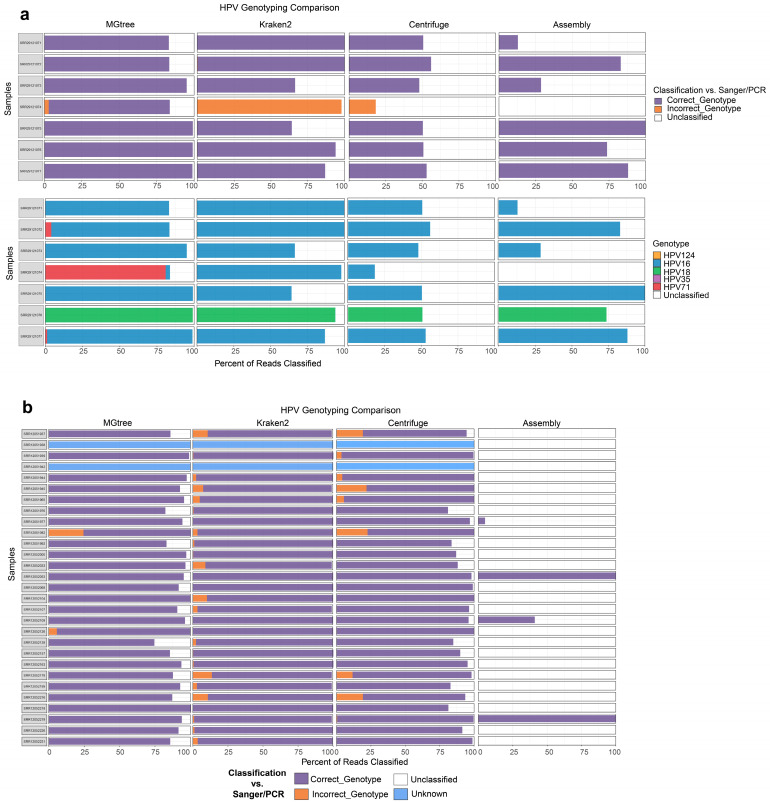
HPV typing by MGtree, Kraken2, Centrifuge, and de novo assembly with metaSPAdes and the PaVE typing tool. (**a**) The percentage of HPV infected cancer cell line reads typed at the genotype level by the four methods are colored by concordance with qPCR (top) and genotype (bottom). For samples with coinfections, reads classified as either of the reported genotypes are considered correct. (**b**) The percentage of HPV cervical cancer reads classified at the genotype level by the four methods are colored by concordance with qPCR.

We benchmarked MGtree on two HPV datasets, including both virally infected cancer cell lines [21], some with known coinfections, and patient-derived cervical cancer samples with low viral titers [22]. With the cancer cell lines, MGtree classified an average of 91.4% of the reads to the correct genotype(s) (Figure 5a). Two of the cell lines have known coinfections, and in both cases, MGtree identified the confirmed genotypes. For one sample, MGtree assigned an incorrect genotype for 2.9% of the reads, but it is possible this cell line also had a coinfection that had not been confirmed. In contrast, Kraken2 and Centrifuge had average correct assignment rates of just 73.0% and 38.6%, respectively, and neither program caught either of the coinfected lines. One sample, SRR29121074, was entirely misassigned by both Kraken2 and Centrifuge, though the assigned genotype matches what we suspect may have been a coinfecting virus. Assembly failed to produce any HPV contigs for this sample, and assembly also did not detect either of the coinfected samples, likely due to the limited number of HPV reads in these samples.

In the cervical cancer samples, MGtree, Kraken2, and Centrifuge all classified over 90% of the reads correctly (Figure 5b). Although Kraken2 classified the most reads correctly, on average, it also displayed a much higher incidence of incorrect assignments; half of the samples analyzed with Kraken2, as well as ten with Centrifuge, had more than 1% of reads misclassified, versus just two samples with MGtree. Due to the relatively low numbers of HPV reads (tens to thousands), assembly was successful on only four samples.

## 4. Discussion

We have demonstrated the superior performance of MGtree as a metagenomics tool, using viral genotyping as example cases. With norovirus samples, MGtree achieved an average genotype-level classification accuracy of 75.3% of reads, compared to just 3.3% with Kraken2 and 17.2% with Centrifuge. In two studies of HPV, MGtree’s accuracy was better than 90%, and it correctly identified the genotypes in coinfected samples.

These results highlight the advantage of MGtree’s alignment-based approach, which makes maximal use of the sequence data. Thus, MGtree is better able to resolve closely related genotypes where k-mer based methods like Kraken2 or variable-length exact match methods like Centrifuge may struggle due to ambiguous or shared short matches. In cases where even a full-length read aligns to multiple reference sequences, MGtree places the read at the LCA node, and it may ultimately be reported as ambiguous if the desired level of classification cannot be discerned by the sequence read itself. This ambiguity is distinct from cases of mixed samples where reads align unambiguously to multiple references. In these cases, MGtree reports the abundance of each appropriately. This transparent reporting of uncertainty is particularly important in viral genotyping where small sequence differences have biological and public-health consequences.

By aligning to reference sequences, MGtree is able to classify even low-abundance samples correctly, which is not the case with de novo assembly. In addition, assembly, even when it succeeds on samples with large numbers of reads, often produces multiple contigs that each contain only part of the genome, which can serve to obfuscate any coinfections or additional lower-abundance taxa in a sample. Assembly is also extremely computationally demanding, and classification via a web-based tool requires manual upload and is less secure.

Another benefit of MGtree’s use of full-length alignment is the potential for additional downstream confirmatory analysis. Users can generate a consensus sequence, similar to a reference-based assembly, for use with any specialized classification websites. Coverage plots and statistics can also be generated to aid in dissecting ambiguous or complicated results (Supplementary Figure S1b). In the future, these enhancements, along with Nextflow support for long-read analysis, could be added to MGtree.

MGtree’s inherent flexibility is a further strength. Users can specify a custom reference database with any number of sequences and taxonomic labels, which allows tailored analyses for any specific or diverse organism populations. References can be easily downloaded from existing databases, such as NCBI, Calicinet, or PaVE, or they can be carefully curated by an expert user down to a precise genomic region of interest. For example, noroviruses can be dual typed—genotyping based on VP1 (ORF2) and P-typing based on RdRp (ORF1)—and one could run MGtree on either, or both. MGtree’s classification in any desired phylogenetic space allows the user to decide on the level of specificity with high precision.

Precise viral genotyping is essential because small genetic differences can alter transmissibility, immune escape, and clinical outcomes, and because public health responses depend on accurate identification of circulating strains. MGtree’s superior classification resolution across the complementary challenges of closely related, highly recombinant, and coinfected viral samples highlight that it could bolster public health and clinical pipelines by improving mapping of circulating diversity, detecting low-frequency genotypes, and reducing false assignments. MGtree also quantifies coinfecting and minority genotypes by accurately reporting samples whose reads map unambiguously to multiple references. Curated, user-defined reference databases let laboratories tailor analyses to local lineages, vaccine strains, or functionally important regions to increase specificity and reduce misclassification. The high-throughput nature of the pipeline allows for automated classification of samples from one or multiple sequencing runs in parallel, allowing for robust sample processing in the case of surveillance or a vaccine clinical trial. Finally, since all the analysis occurs in a closed system, MGtree is optimal for proprietary and clinical samples. Together, these capabilities make MGtree well-suited for both routine surveillance and rapid-response frameworks, and its combination of high accuracy, adaptability, and explicit uncertainty handling helps ensure genotype calls are actionable and interpretable.

## 5. Conclusions

MGtree is a robust pipeline that allows for accurate metagenomics analysis of NGS data. Metagenomics is a rapidly expanding field that includes diverse applications, and traditional orthogonal methods such as PCR and Sanger sequencing, though highly accurate, are constrained by lower throughput and a limited ability to detect multiple or novel genotypes simultaneously. NGS enables high-throughput, comprehensive profiling of entire microbial communities, capturing both abundant and rare organisms in a single assay. MGtree provides a flexible and powerful platform for NGS data analysis with several key advantages. While our benchmarking and initial application focused on viral datasets, MGtree’s design and flexibility make it broadly applicable to any metagenomics study, supporting the growing need for precise and adaptable analytical methods.

## Data Availability

MGtree consists of Python scripts, wrapped in Nextflow along with open-source software. It is freely available at https://github.com/MSDLLCpapers/MGtree. The datasets analyzed in this work are publicly available on NCBI SRA and are detailed in Table S1.

## Supplementary Materials

The following supporting information can be downloaded at: https://www.mdpi.com/article/10.3390/v18060643/s1, Figure S1: Norovirus typing by MGtree, Kraken2, Centrifuge, and de novo assembly with metaSPAdes and the Calicivirus typing tool; Table S1: SRA sample IDs for norovirus and HPV benchmarking; Table S2: CaliciNet NCBI accession IDs for norovirus reference genomes; Table S3: Computational requirements of MGtree, Kraken2, Centrifuge, and a de novo assembly approach for the processing of norovirus samples; Supplementary Note S1: MGtree implementation details; Supplementary Note S2: Benchmarking details.

## Abbreviations

The following abbreviations are used in this manuscript:

NGS	Next-generation sequencing
HPV	Human papillomavirus
LCA	Lowest common ancestor
WGS	Whole genome sequenced
RT-PCR	Reverse transcription polymerase chain reaction
qPCR	Quantitative polymerase chain reaction
