# Supplementary material for: MGtree: A Fast and Flexible Alignment-Based Metagenomics Pipeline
Source: Viruses. 2026 Jun 3;18(6):643. doi: 10.3390/v18060643 (PMC13307765; doi:10.3390/v18060643)
Supplement: Supplementary file 1 [file viruses-18-00643-s001.zip › viruses-4308135-supplementary.pdf]

MGtree: A fast and flexible alignment-based metagenomics pipeline

Samantha L. Sholes, Scott Norton, Alfredo Gonzalez, John M. Gaspar

Supplementary Information

Contents

1. Supplementary Figure
    a. Figure S1: Norovirus typing by MGtree, Kraken2, Centrifuge, and *de novo* assembly with metaSPAdes and the Calicivirus typing tool
2. Supplementary Tables
    a. Table S1: SRA sample IDs for norovirus and HPV benchmarking
    b. Table S2: CaliciNet NCBI accession IDs for norovirus reference genomes
    c. Table S3: Computational requirements of MGtree, Kraken2, Centrifuge, and a *de novo* assembly approach for the processing of norovirus samples
3. Supplementary Note S1: MGtree implementation details
4. Supplementary Note S2: Benchmarking details
5. Supplementary References

Supplementary Figure

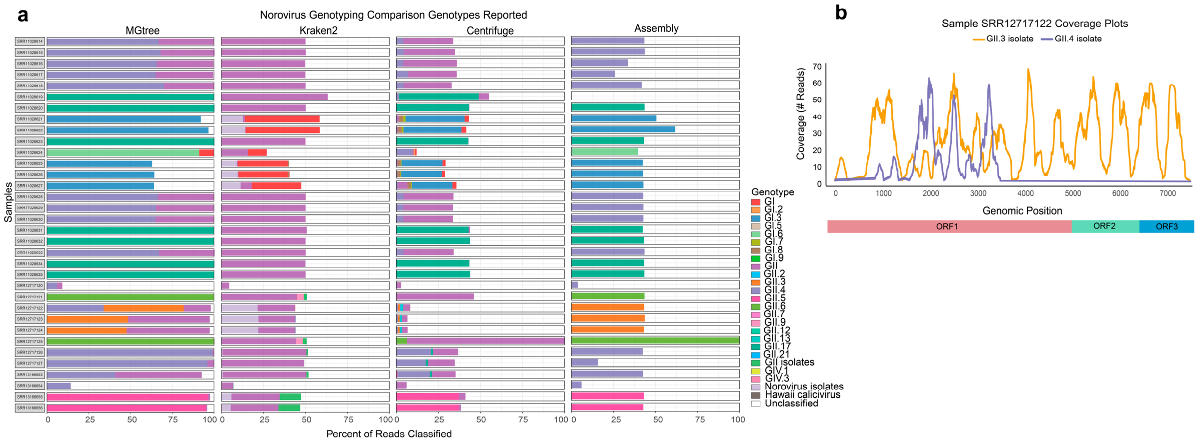

Figure S1: Norovirus typing by MGtree, Kraken2, Centrifuge, and *de novo* assembly with metaSPAdes and the Calicivirus typing tool. **a)** The percentage of norovirus WGS stool sample reads typed at the genotype and genogroup level by the four methods are colored by genotype and genogroup reported. Note that the assembly approach is classified only to the genotype level. **b)** MGtree coverage plots for sample SRR12717122. The correct (by PCR) reference genome, GII.3, has reads across the entire norovirus genome, including ORF2. The incorrect (by PCR) reference genome, GII.4, has reads in the ORF1 region only.

Table S1. The SRA study and individual SRA sample IDs used in norovirus and HPV analysis. Samples with genotype confirmation by PCR or Sanger Sequencing were chosen for benchmarking in this study.

Dataset	Norovirus [1]	HPV[2]	HPV [3]
**SRA study**	SRP247410	SRP509119	SRP220229
**SRA sample IDs**	SRR11028614	SRR29121071	SRR12051937
	SRR11028615	SRR29121072	SRR12051938
	SRR11028616	SRR29121073	SRR12051939
	SRR11028617	SRR29121074	SRR12051942
	SRR11028618	SRR29121075	SRR12051944
	SRR11028619	SRR29121076	SRR12051945
	SRR11028620	SRR29121077	SRR12051965
	SRR11028621		SRR12051976
	SRR11028622		SRR12051977
	SRR11028623		SRR12051982
	SRR11028624		SRR12051992
	SRR11028625		SRR12052000
	SRR11028626		SRR12052033
	SRR11028627		SRR12052053
	SRR11028628		SRR12052068
	SRR11028629		SRR12052104
	SRR11028630		SRR12052107
	SRR11028631		SRR12052109
	SRR11028632		SRR12052126
	SRR11028633		SRR12052139
	SRR11028634		SRR12052157
	SRR11028635		SRR12052163
	SRR12717120		SRR12052178
	SRR12717121		SRR12052199
	SRR12717122		SRR12052216
	SRR12717123		SRR12052218
	SRR12717124		SRR12052219
	SRR12717125		SRR12052226
	SRR12717126		SRR12052231
	SRR12717127		
	SRR13168653		
	SRR13168654		
	SRR13168655		
	SRR13168656		

Table S2. The NCBI accession IDs from the custom collection of 204 norovirus reference genomes with complete VP1 sequences (genotyping region) from the CDC CaliciNet database [4].

MH130046.1	DQ379715.1	AY823305.2	AF414427.1	AB126320.1
EU085529.1	DQ456824.1	AY823306.1	KJ196290.1	EF126965.1
KP027330.1	AB083780.1	DQ078814.2	GQ856474.1	EF529741.1
KJ196298.1	AB220921.1	AB190457.1	OR262322.1	EF547396.1
HQ637267.1	AB220922.1	AY502009.1	AJ004864.1	EU078417.1
GQ856463.1	AB983218.1	AY502010.1	AF145709.1	AB294790.1
GQ856464.1	KJ196276.1	AY237415.2	AF195847.1	EU373815.1
GQ856470.1	KJ196277.1	AY502023.1	AF195848.1	AB434770.1
GQ856471.1	KJ196278.1	AY134748.1	AJ277607.1	AB281090.1
GQ856472.1	KJ196284.1	AF539439.1	AJ277608.1	AB385626.1
GQ856473.1	KJ196286.1	AB039775.1	AJ277609.1	AB447406.1
GQ856475.1	KJ196288.1	AB039777.1	AJ277614.1	GQ856465.1
L23828.1	KJ196295.1	AB039778.1	AJ277618.1	EU921353.2
U04469.1	KM198534.1	AB039780.1	AJ277620.1	EU921354.2
M87661.2	GQ856469.1	AF414409.1	AF394960.1	GU594162.1
L07418.1	U07611.2	AF414410.1	AF425767.1	AB542917.1
KJ402295.1	U02030.1	AF414424.1	AF427118.1	HM633213.1
KF586507.1	AB684728.2	AY038599.2	AF435807.1	JF697282.1
FJ515294.1	KU306738.1	AF190817.1	AF439267.1	JN565063.1
LC342057.1	KJ194500.1	X81879.1	AB074893.1	JN183159.1
LN854563.1	KJ194507.1	U22498.1	AY113106.1	JQ970479.1
JN899243.1	AB541348.1	MK733205.1	AY130761.1	KC576910.1
AB187514.1	GU930737.1	MN400355.2	AY130762.1	KJ196292.1
AB039774.1	GU445325.2	MK762630.1	AF538678.1	KM289169.1
AB081723.2	GU017903.2	MG706448.1	AF538679.1	KM289171.1
AF414406.1	FJ537134.1	MG495079.1	AF542090.1	LC101824.1
AY038598.1	FJ537135.1	MG495080.1	AY485642.1	LC101825.1
AJ313030.1	GQ845367.2	MG495082.1	AB112306.1	KT239614.1
AB042808.1	GQ845370.2	MG495083.1	AY502006.1	LC153121.1
AF093797.1	EU921388.2	MG495084.1	AY675554.1	KT030674.1
KU311161.1	MW559992.1	KT290889.1	AY675555.1	KX353972.1
MH443711.1	MW019958.1	KT589391.1	AY682550.1	KX354134.1
MT028542.1	MK752934.1	MH218579.1	AY682552.1	KX061540.1
JX989075.1	LC342059.1	MH218692.1	AY772730.1	KY225989.1
JX846924.1	MF140689.1	KX158281.1	AY883096.1	MF352142.1
JX846925.1	MF405169.1	KC894731.1	DQ093067.1	MN248516.1
JX459907.1	AB933767.1	KX907728.1	AJ844469.1	MW521126.1
JX459908.1	LC037415.1	FM865412.1	AJ844470.1	OL336352.1
EU424333.1	JN797508.1	JQ613567.1	DQ438972.1	AB684696.2
DQ379714.1	AY823304.1	AF414426.1	AB220926.1	OR262344.1
AJ277615.1	EF126963.1	AB985418.2	KJ196291.1	

Table S3. Computational requirements of MGtree, Kraken2, Centrifuge, and a *de novo* assembly approach for the processing of norovirus samples. For database generation with MGtree, labeling the Newick string is included. Sample processing with the assembly approach includes removal of human reads with bowtie2 and *de novo* assembly with metaSPAdes, but does not include the manual uploading of assembled contigs to web-based software for classification.

		**MGtree**	**Kraken2**	**Centrifuge**	**Assembly**
**Database generation**	**CPU hours**	0.0167	1.8	0.868	-
	**Memory (GB)**	0.161	7.0	0.472	-
	**Disk space (MB)**	0.97	49104	8950	-
**Sample processing**	**CPU hours**	1.52	0.355	0.584	236236
	**Memory (GB)**	0.432	0.0426	0.0287	11.1
	**Disk space (MB)**	0.25	4200	4260000	12000

Supplementary Note S1: MGtree implementation details

MGtree performs metagenomics classification of reads against a set of reference sequences via a phylogenetic tree. It is alignment-based, making it particularly suitable for querying against references that are very similar to each other.

The pipeline produces a list of the reference genotypes and relative abundances that are found in a given sample. Note that the pipeline, and this manual, always refer to the output classifications as genotypes, but the output could be at any of a number of levels (e.g. serotype, species, strain, etc.), depending on the reference sequences that are being queried against and the desired level of precision.

Reference sequences

Reference sequences should be collected in a single fasta-formatted file. They will be used to create a phylogenetic tree, and a reference index against which to align the reads. These procedures need to be done only once for a given set of reference sequences, even if multiple samples are to be processed against them.

Creating a phylogenetic tree

Before running `MGtree.py`, a phylogenetic tree must be created from the reference sequences to be queried. This can be accomplished by software such as MEGA-X [5]. The tree must be exported as a Newick string for use by the rest of the pipeline.

Label tree nodes (updateNewick.py)

This script reads in a Newick string from an input file and outputs a labeled Newick string. It gives every unnamed node a unique name (an integer), and it can assign genotypes to nodes that are listed in an optional input csv file.

Reference indexing

To align reads to a set of references, the reference sequences must be indexed. The Nextflow wrapper of our pipeline uses the short read aligner `bowtie2` [6], whose indexes are created by `bowtie2-build`.

Sample processing

The rest of the MGtree pipeline analyzes a single sample and produces a list of the reference genotypes and relative abundances found therein. It requires the phylogenetic tree and reference index previously created. The pipeline is designed to analyze a sample that has been sequenced via some type of massively parallel sequencing, such as Illumina. The sequencing can be targeted or shotgun, and single-end or paired-end.

The pipeline comes with a Nextflow wrapper for the convenient and robust batch processing of a set of samples. The Nextflow wrapper contains the option to pre-process and align (using `bowtie2`) the samples as part of the MGtree pipeline. Both the scripts for processing an individual sample (main branch) and the Nextflow wrapper for batch processing (nextflow branch) are available in the GitHub repository.

Read alignment

As stated above, this pipeline is particularly suited for querying against references that are very similar. To this end, it works best when *all* valid alignments of a read/fragment are available to be analyzed. This can be accomplished with our preferred aligner, `bowtie2` [6] in `-a` mode.

After alignment, the resulting SAM file is expected to be sorted by queryname. The sorting can be accomplished via `samtools sort -n`, although that extra step can be avoided if the SAM file produced directly by `bowtie2` is left unmanipulated (conversion to BAM is fine).

Alignment parsing (parseSAM.py)

This script analyzes a SAM file, considering primary alignments of reads/fragments, as well as secondary alignments that are equivalent (or within a specified threshold, based on alignment scores). For paired-end sequencing, if the primary alignment is in a properly paired configuration, only secondary alignments that are also properly paired are evaluated. The script produces a file that lists, for each read/fragment, the alignment type and the reference sequence(s) to which it aligns.

Metagenomics classification (MGtree.py)

This script analyzes a file of parsed alignments and a phylogenetic tree via a Newick string. For each read/fragment, it adds counts to the tree at the lowest common ancestor (LCA) node of the set of alignments. Properly paired alignments are given a weight of two, and unpaired alignments are each counted as one, as are alignments for single-end reads. Once the alignments are added to the tree, the script interprets the LCA counts for nodes with assigned genotypes, providing a classification profile of the sample. Depending on the taxonomic level of interest, the user can choose to set the `-g` option, in which case the leaf nodes will *not* have genotypes interpreted in the classification profile.

The primary output of `MGtree.py` is a sorted list of the genotypes observed in the sample, along with the read counts and percentages. LCA counts at a node that is an ancestor of multiple genotype nodes will be reported as ambiguous, such as `ambig[GII.4,GII.5]`. If no descendant genotypes can be found, the result will be reported as `ambig[?]`. There are optional output files, including one listing counts at leaf nodes (corresponding to the original reference sequences used to build the tree), which is useful for identifying the most closely related reference sequence for additional coverage verification or variant analysis.

Testing

The repository for MGtree includes a `test.sh` script to test the pipeline systematically using test files located in the `test/` folder.

Supplementary Note S2: Benchmarking details

This section details how MGtree v1.0, Kraken2 v2.1.2 [7], Centrifuge v1.0.4.2 [8], and metaSPAdes v4.2.0 [9] combined with Genome Detective [10], the Calicivirus typing tool [11], and the PaVE BLAST typing tool [12], were invoked for the benchmark tests.

Reference database generation

For the analysis of norovirus samples, we generated a custom database containing 204 norovirus isolate reference sequences from the CDC CaliciNet database of complete (VP1 regions), confirmed, and genotyped norovirus sequences [4] (Table S2). Note that this reference is included in the `test/` folder of the GitHub repository.

To generate the MGtree database, we loaded the reference fasta file into MEGA-X and chose phylogeny, Construct Neighbor-Joining Tree. Once constructed, the tree was output as a Newick file including branch lengths.

We labeled the tree nodes with unique IDs:

```
$ updateNewick.py -i NoV_database.nwk -o NoV_database_id.nwk
```

We manually determined which nodes to label with genotype or genogroup designations, collected these annotations in a .csv file, and then updated the tree:

```
$ updateNewick.py -i NoV_database_id.nwk -o NoV_database_labeled.nwk
-n node_labels.csv
```

We built the bowtie2 reference index for the reference fasta file:

```
$ bowtie2-build reference.fa referenceIdx
```

The reference fasta file, bowtie2 index referenceIdx, and NoV_database_labeled.nwk constitute the reference database for MGtree.

To generate the kraken2 custom database, we ran the following commands:

```
$ kraken2-build --download-taxonomy --db NoV_database

$ kraken2-build --add-to-library reference.fa --db NoV_database

$ kraken2-build --build --db NoV_database
```

To generate the Centrifuge custom database, we first downloaded the taxonomy from NCBI, extracted the NCBI taxid from each reference accession to build the seqid.taxid.map, then ran:

```
$ centrifuge-build --conversion-table seqid_taxid.map --taxonomy-tree
taxonomy/nodes.dmp --name-table taxonomy/names.dmp NoV_reference.fasta
NoV_database
```

For HPV, we downloaded 224 HPV reference genomes from the PAVE database [13] and repeated the above process.

Preprocessing, alignment, and multimapping processing

Norovirus samples with confirmed genotypes via orthogonal methods of PCR or Sanger Sequencing were downloaded from SRA (Table S1).

We preprocessed and filtered the datasets with FastQC v0.11.9 [14] and fastp v1.0.0 [15]:

```
$ fastqc Sample1_R1.fastq.gz Sample1_R2.fastq.gz
```

```
$ fastp -i Sample1_R1.fastq.gz -I Sample1_R2.fastq.gz
-o Sample1_R1_processed.fastq.gz -O Sample1_R2_processed.fastq.gz
-q 15 -l 25
```

Filtered reads were aligned to the norovirus reference database with `bowtie2` v2.3.5.1 [6] with `-a` to retain all alignments, and `parseSAM.py` was run in default mode to parse the alignments:

```
$ bowtie2 -a -x reference_index -1 Sample1_R1_processed.fastq.gz
-2 Sample1_R2_processed.fastq.gz -S | samtools sort -n
-o Sample1_name_sorted.sam
```

```
$ parseSAM.py -i Sample1.name_sorted.sam -o Sample1_parsed.tsv
```

Classification

We classified the reads with MGtree, specifying `-g` to ignore leaf nodes, since there were multiple references per genotype:

```
$ MGtree.py -i Sample1_parsed.tsv -n NoV_database_labeled.nwk
-o Sample1_genotypes.tsv -t Sample1_leafnodes.tsv -g
```

We classified the reads with Kraken2 via the following command:

```
$ kraken2 --report Sample1_report.txt --db Norovirus_database/
--output Sample1_output.txt --report-minimizer-data --paired
Sample1_R1.fastq.gz Sample1_R2.fastq.gz
```

We classified the reads with Centrifuge with the following command:

```
$ centrifuge -x Norovirus_database -1 Sample1_R1.fastq.gz
-2 Sample1_R2.fastq.gz --min-hitlen 50 -S Sample1_report.tsv
```

The processing of HPV samples was similar, except that MGtree.py was run without `-g`.

The read counts at each taxonomic level were then divided by the total number of norovirus or HPV reads reported in each published dataset to calculate the percentage of reads classified per sample. A read was considered correctly classified if it matched the genotype (or genogroup) defined by PCR or Sanger Sequencing in the published results.

Assembly

For norovirus datasets, we performed viral assembly with metaSPAdes [9]. First, we first identified human-derived reads from the samples using bowtie2 [6]:

```
$ bowtie2 -x hg38 -1 Sample1_R1_processed.fastq.gz
-2 Sample1_R2_processed.fastq.gz --un-conc Sample1.unmapped.fastq.gz
-S Sample1.sam
```

Then, we assembled the unmapped (non-human) reads with metaSPAdes using the `--metaviral` setting:

```
$ spades.py -1 Sample1.unmapped.1.fastq.gz
-2 Sample1.unmapped.2.fastq.gz -o Sample1_assembly_output --metaviral
```

For samples where no norovirus contigs were produced, we transitioned to the less stringent `--meta` setting:

```
$ spades.py -1 Sample1.unmapped.1.fastq.gz
-2 Sample1.unmapped.2.fastq.gz -o Sample1_assembly_output --meta
```

Finally, we classified the norovirus assemblies with Genome Detective [10] and the Calicivirus typing tool [11] web browsers. Since the assembly results contained tens to thousands of contigs, we selected the first twenty contigs (ordered by size) to load into the websites, due to fasta import size limits. If no contigs were reported as norovirus, we continued in twenty-contig intervals until all contigs were tested. The genotype was recorded for one sample at a time from the typing website if a resulting contig was reported as norovirus. The contig size and coverage was recorded from the fasta header. The read counts supporting each contig were calculated via this formula:

```
read counts = ((contig size * contig coverage) / read length)
```

The assembly of HPV samples was similar, except that the contigs were classified using the PaVE BLAST typing tool [12].

Supplementary References

1. Silva, A.J.; Yang, Z.; Wolfe, J.; Hirneisen, K.A.; Ruelle, S.B.; Torres, A.; Williams-Hill, D.; Kulka, M.; Hellberg, R.S. Application of whole-genome sequencing for norovirus outbreak tracking and surveillance efforts in Orange County, CA. *Food Microbiol* **2021**, *98*, 103796, doi:10.1016/j.fm.2021.103796.
2. Shen-Gunther, J.; Easley, A. HPV, HBV, and HIV-1 Viral Integration Site Mapping: A Streamlined Workflow from NGS to Genomic Insights of Carcinogenesis. *Viruses* **2024**, *16*, doi:10.3390/v16060975.
3. Arroyo Muhr, L.S.; Lagheden, C.; Eklund, C.; Lei, J.; Nordqvist-Kleppe, S.; Sparen, P.; Sundstrom, K.; Dillner, J. Sequencing detects human papillomavirus in some apparently HPV-negative invasive cervical cancers. *J Gen Virol* **2020**, *101*, 265–270, doi:10.1099/jgv.0.001374.
4. CaliciNet. Available online: https://www.cdc.gov/norovirus/php/reporting/calicinet.html (accessed on 27 August 2025).
5. Kumar, S.; Stecher, G.; Li, M.; Knyaz, C.; Tamura, K. MEGA X: Molecular Evolutionary Genetics Analysis across Computing Platforms. *Mol Biol Evol* **2018**, *35*, 1547–1549, doi:10.1093/molbev/msy096.
6. Langmead, B.; Salzberg, S.L. Fast gapped-read alignment with Bowtie 2. *Nat Methods* **2012**, *9*, 357–359, doi:10.1038/nmeth.1923.
7. Wood, D.E.; Lu, J.; Langmead, B. Improved metagenomic analysis with Kraken 2. *Genome Biol* **2019**, *20*, 257, doi:10.1186/s13059-019-1891-0.
8. Kim, D.; Song, L.; Breitwieser, F.P.; Salzberg, S.L. Centrifuge: rapid and sensitive classification of metagenomic sequences. *Genome Res* **2016**, *26*, 1721–1729, doi:10.1101/gr.210641.116.
9. Nurk, S.; Meleshko, D.; Korobeynikov, A.; Pevzner, P.A. metaSPAdes: a new versatile metagenomic assembler. *Genome Res* **2017**, *27*, 824–834, doi:10.1101/gr.213959.116.
10. Vilsker, M.; Moosa, Y.; Nooij, S.; Fonseca, V.; Ghysens, Y.; Dumon, K.; Pauwels, R.; Alcantara, L.C.; Vanden Eynden, E.; Vandamme, A.M.; et al. Genome Detective: an automated system for virus identification from high-throughput sequencing data. *Bioinformatics* **2019**, *35*, 871–873, doi:10.1093/bioinformatics/bty695.
11. Tatusov, R.L.; Chhabra, P.; Diez-Valcarce, M.; Barclay, L.; Cannon, J.L.; Vinje, J. Human Calicivirus Typing tool: A web-based tool for genotyping human norovirus and sapovirus sequences. *J Clin Virol* **2021**, *134*, 104718, doi:10.1016/j.jcv.2020.104718.
12. Van Doorslaer, K.; Li, Z.; Xirasagar, S.; Maes, P.; Kaminsky, D.; Liou, D.; Sun, Q.; Kaur, R.; Huyen, Y.; McBride, A.A. The Papillomavirus Episteme: a major update to the papillomavirus sequence database. *Nucleic Acids Res* **2017**, *45*, D499–D506, doi:10.1093/nar/gkw879.
13. Reference genomes for Human papillomavirus. Available online: https://pave.niaid.nih.gov/explore/reference_genomes/human_genomes (accessed on 27 August 2025.
14. Brown, J.; Pirrung, M.; McCue, L.A. FQC Dashboard: integrates FastQC results into a web-based, interactive, and extensible FASTQ quality control tool. *Bioinformatics* **2017**, *33*, 3137–3139, doi:10.1093/bioinformatics/btx373.
15. Chen, S.; Zhou, Y.; Chen, Y.; Gu, J. fastp: an ultra-fast all-in-one FASTQ preprocessor. *Bioinformatics* **2018**, *34*, i884–i890, doi:10.1093/bioinformatics/bty560.
